# Populations of doubled haploids for genetic mapping in hexaploid winter triticale

**DOI:** 10.1007/s11032-018-0804-3

**Published:** 2018-03-30

**Authors:** M. Tyrka, S. Oleszczuk, J. Rabiza-Swider, H. Wos, M. Wedzony, J. Zimny, A. Ponitka, A. Ślusarkiewicz-Jarzina, R. J. Metzger, P. S. Baenziger, A. J. Lukaszewski

**Affiliations:** 1 Department of Biotechnology and Bioinformatics, Rzeszow University of Technology, Rzeszow, Poland; 2 Institute of Plant Breeding and Acclimatization, National Research Institute, Radzikow, Poland; 3 Department of Ornamental Plants, Warsaw University of Life Sciences, Warsaw, Poland; 4 Plant Breeding Strzelce Ltd., Co. - IHAR-PIB Group, Strzelce, Poland; 5 Department of Cell Biology and Genetics, Pedagogical University of Cracow, Kraków, Poland; 6 Institute of Plant Genetics, Poznan, Poland; 7 Department of Crop and Soil Science, Oregon State University, Corvallis, OR 97331-3002 USA; 8 Department of Agronomy and Horticulture, University of Nebraska, Lincoln, NE USA; 9 Department of Botany and Plant Sciences, University of California, Riverside, CA 92521 USA

**Keywords:** Consensus map, DArT markers, Double haploid lines, Triticale, Vernalization, QTL

## Abstract

To create a framework for genetic dissection of hexaploid triticale, six populations of doubled haploid (DH) lines were developed from pairwise hybrids of high-yielding winter triticale cultivars. The six populations comprise between 97 and 231 genotyped DH lines each, totaling 957 DH lines. A consensus genetic map spans 4593.9 cM is composed of 1576 unique DArT markers. The maps reveal several structural rearrangements in triticale genomes. In preliminary tests of the populations and maps, markers specific to wheat segments of the engineered rye chromosome 1R (RM1B) were identified. Example QTL mapping of days to heading in cv. Krakowiak revealed loci on chromosomes 2BL and 2R responsible for extended vernalization requirement, and candidate genes were identified. The material is available to all parties interested in triticale genetics.

**Electronic supplementary material:**

The online version of this article (10.1007/s11032-018-0804-3) contains supplementary material, which is available to authorized users.

## Introduction

Hexaploid triticale (×*Triticosecale* Wittm.), a wheat-rye hybrid with genomic constitution 2*n* = 6*x* = 42 (AABBRR) is slowly becoming an important cereal, already cultivated on about 4 million ha worldwide with over 70% of this area concentrated in the European Union (FAOSTAT 2014). Commercial importance of triticale is justified as it combines favorable agronomic characteristics of both parents, such as high yield potential and good grain quality of wheat with good adaptation to demanding growing conditions of rye.

In breeding, triticale is treated as a self-pollinating species even if cross pollination is relatively frequent (Sowa and Krysiak 1996; Gulmezoglu 2004). Common breeding approaches include standard or modified techniques for self-pollinators, such as single-seed descend, the pedigree method, and others. In most cases, selection is phenotypic, which leaves triticale somewhat behind modern trends and fashions in major crops, threading towards DNA-based approaches. Breeding objectives of triticale focus on grain yield, adaptation towards biotic and abiotic stresses (Tyrka and Chełkowski 2004; Niedziela et al. 2012), and development of hybrid cultivars (Góral 2002; Oettler et al. 2003, 2005; Tams et al. 2006; Stojałowski et al. 2013). Recently, triticale has also been considered as bioenergy crop (Oettler et al. 2003; Pronyk and Mazza 2011) and some consideration is given to the improvement of the bread-making properties (Lukaszewski 2006; Wos and Brzezinski 2015).

The practical value of molecular markers for crop improvement is well-established. In marker-assisted selection, DNA markers are useful for pyramidization of disease resistance genes, controlling fertility restoration, and other agronomic traits (Liu et al. 2012; Zuo and Li 2014). The rationale behind the push for the application of DNA markers in breeding is the expectation that identification of markers linked or located within genes affecting agronomic traits will improve selection efficiency and perhaps even move the entire process of cultivar development from the field to enclosed and automated spaces. The first and indispensable step towards this goal is the creation of high-density reference genetic maps of entire genomes. Such maps provide a useful resource for gene mapping, for linking physical and genetic maps, comparative genomics, and for predicting breeding and genetic values for traits of agricultural significance (Crossa et al. 2007; Saintenac et al. 2013; Raman et al. 2014; Ren et al. 2015). High-density maps have been created for most crops, as well as many non-crop species including triticale (Alheit et al. 2011; Tyrka et al. 2011, 2015). The development of species-specific microarrays and genotyping by sequencing technology enabled application of genomic selection in plant breeding programs (Desta and Ortiz 2014). If triticale is to benefit from the current trends, a reliable consensus map has to be created, preferably in stocks that can readily be used by others for reference, verification, and further development. This requires sets of mapping populations.

Dense genetic maps based on DNA markers can be generated using several different techniques/approaches, and each one has certain advantages and problems. The main advantage of the DArT system (Jaccoud et al. 2001) is that it requires no DNA sequence information and can be generated quickly, and relatively cheaply, for any target. DArT marker sets and DArT-based genetic maps have been developed for many crop species (current list at http://www.diversityarrays.com/) including triticale parents, wheat (Akbari et al. 2006), and rye (Bolibok-Brągoszewska et al. 2009). They have also been tested in triticale (Badea et al. 2011).

The effort described in this article was undertaken to create a shared resource in the form of a set of mapping populations of doubled haploids of hexaploid winter triticale, each one with its DArT-based genetic map, for general use of all interested parties. Along the way, an attempt was made to analyze the extent of segregation distortion and identification of regions with structural rearrangements in respect to previously published genetic maps of triticale. It is hoped that a similar group effort at phenotyping these mapping populations will bring about a better understanding of the triticale genome, will localize in the major quantitative trait loci responsible for its agronomic characteristics, and perhaps shed some light on the concerted evolution of parental genomes in a newly formed amphiploid.

## Materials and methods

### Plant material

The starting materials were four cultivars and one breeding line of hexaploid winter triticale originating from various breeding programs. All are (or were in their prime) high yielding under conditions for which they were developed. The entries were cv. Presto, obtained as a breeding line CT775/81 (abbreviated as CT) from the late Dr. E. Tymieniecka, then Poznanska Hodowla Roslin (now Hodowla Roslin Danko, Choryn, Poland); cv. Mungis (MU), obtained from Dr. B. Schinkel, then Lochow-Petkus GmbH, Bergen, Germany (now KWS); cv. Krakowiak (KR), contributed by Dr. H. Wos, then at the Strzelce Plant Breeding, Borowo, Poland; cv. Stan 1 (ST), obtained from the late Dr. S. Nalepa, then at the Resource Seed Inc., Gilroy, California, USA; and a breeding line NE422T (NE) contributed by Dr. P.S. Baenziger, University of Nebraska, Lincoln, NE, USA. Presto (CT) was one of the early and very successful winter triticales released all over Europe; it is still grown on a considerable acreage and is used by Adam Lukaszewski (AJL) as the repository of all chromosome constructs; “Mungis” (MU) was selected for its high and stable falling number in official registration tests in Poland; “Krakowiak” (KR) was selected for its good winter hardiness and high falling number, Stan 1 (ST) for good adaptation in several areas of the USA, and NE422T (NE) represented a high forage/biomass cultivar adapted to dryland regions of the Great Plains in the USA. In crosses with Krakowiak, Mungis, and NE422T, Presto was represented by two sub-lines: standard Presto and Presto RM1B. Presto RM1B carries engineered chromosome 1R (Lukaszewski, 2006) which is a six-breakpoint translocation: on the short arm, it has one segment from chromosome 1BS of cv. Pavon 76 that introduces loci *Gli-B1/Glu-B3* and another segment which removes rye locus *Sec-1*; on the long arm, it has a segment from wheat chromosome 1DL carrying high-molecular-weight glutenin locus *Glu-D1* originating from cv. Wheaton. This segment replaces a segment of 1R carrying locus *Sec-3*.

The parental lines were crossed pairwise, and the resulting F_1_ hybrids were used to generate doubled-haploid progeny populations via androgenesis. Individual F_1_ hybrids are designated by the combination of the parental acronyms, and so the Mungis × Krakowiak F_1_ is referred to as MUKR and all doubled haploids derived from it are designated by the same acronym followed by a number identifying individual lines. The original plan called for a scheme where each parental line was to be present in three different cross combinations, but it quickly turned out too ambitious. Eventually, six populations of doubled haploids were generated, totaling 1238 lines; after the removal of clones and aneuploids, the total number of DH lines was reduced to 957, with population sizes ranging from 97 to 231 (Table 1 and Online resource 1-6). Respective subpopulations of MUCT, KRCT, and NECT were screened for high-molecular-weight glutenins and their corresponding *Sec-3* secalins by SDS-PAGE (Brzezinski and Lukaszewski 1998).

**Table 1:** Description of the mapping populations

Population code	Pedigree	Effective size^a^	Redundant and aneuploid lines	DArT markers
MUKR	“Mungis” × “Krakowiak”	144	75	1198
MUST	Mungis × “Stan 1”	172	25	1237
MUCT	Mungis × “Presto”	231	123	1507
KRST	Krakowiak × Stan 1	138	23	996
KRCT	Krakowiak × Presto	175	21	1048
NECT	“NE422T” × Presto	97	7	2041

^a^Size of mapping population composed of unique genotypes

^b^Initial number of markers after quality selection

### Doubled haploid production

Individual populations, or parts of some populations, were created by androgenesis in laboratories of individual co-authors. The protocols used in different laboratories were similar, with local modifications and adaptations. The general protocol was the same as in Oleszczuk et al. (2011). Tillers with anthers at about the late uninucleate stage were cut from greenhouse-grown F_1_ hybrids and stored in the dark in water at ca. 3–6 °C for 2 to 4 weeks. In an alternative method, used extensively at UCR, stems were kept in the N6 solution supplemented with 2,4D (Ryöppy 1997) for 2–4 days, also at 3–6 °C. AJL often moved collected heads from water to N6 and vice versa, depending on plating schedule. Immature heads were extracted from such stored and aged material, surface sterilized in 70% ethanol and 6% sodium hypochlorite, anthers dissected and plated on the C17 medium (Wang and Chen 1983), and stored in the dark at room temperature for up to 3 months. Developed embryogenic structures were transferred onto the 190–2 medium (Zhuang and Xu 1983), transferred to light, and eventually transplanted to soil.

For doubling of the chromosome number, all plants were treated at the tillering stage with aerated 0.1% solution of colchicine in water, with 3% DMSO, for 7–8 h, rinsed in water overnight, transplanted to soil and then vernalized for about 8 weeks at ca. 3–7 °C. All emerging heads were bagged to avoid cross pollination. The resulting lines were increased once in the greenhouse, again with bagging of all heads, and planted in the field for a large seed increase. Samples originating from the greenhouse grown material are available from AJL; the field-grown material is currently with Dr. J. Larsen, Agri-Food Canada, Lethbridge, Alberta.

### DArT and data analyses

Total genomic DNA was isolated from fragments of young leaves from each genotype, frozen at − 80 °C and crushed in liquid nitrogen with the aid of steel pellets using the Qiagen shaker. DNA extraction and handling followed the protocol recommended for the DArT procedure (see http://www.diversityarrays.com/submission-sample). DNA integrity was tested on agarose gels, while its quantity was measured spectrophotometrically. DNA samples were submitted to the Diversity Array Technology (Yarralumla, Australia) for profiling using their triticale high-resolution array (DArT) with 7296 probes representing markers from wheat, rye, and triticale (wPt, rPt, and tPt, respectively). Cluster and principal component analyses based on Dice similarity indices were applied to study relationships between the parental lines using PAST (Hammer et al. 2001).

### Construction of genetic linkage maps

De novo mapping approach was used to construct genetic maps for individual segregating populations. Markers of unknown parental origin and present at frequency 95% < F < 5% were removed from the dataset. To simplify calculations, DArT markers were binned with quantitative trait loci (QTL) IciMapping (Wang et al. 2016) and representative markers with the lowest number of missing data were left to represent bins. Segregation data were analyzed with JoinMap 4 (Van Ooijen 2006) to group markers using the logarithm of odds (LOD) > 3. Markers within these groups were recurrently ordered using the JoinMap maximum likelihood option and the RECORD program (Van Os et al. 2005). Group length and the maximum expected number of recombination events per individual were the criteria for selecting marker order for the next ordering cycle. Optimal marker order was used to sort markers within linkage groups, and graphical genotypes were examined in Excel 2013. At this step, singletons (a single locus from one parent appearing in a block of loci from the other parent) were replaced by missing values in the dataset and calculations were repeated until no singletons were found (no more than three rounds). Consensus genetic map was constructed using the MergeMap online tool (http://www.mergemap.org/, Wu et al. 2011). The resulting marker order corresponding to chromosomes and chromosome segments was compared with the triticale map consensus developed for a different set of genotypes (Alheit et al. 2011) and “Saka3006” × “Modus” (SM, Tyrka et al. 2011).

### QTL analysis

Vernalization requirements were tested for the KRST population at the University of California, Riverside. Several seeds from each DH line and the parents were planted in flats, germinated for 24 h at room temperature, and transferred to a vernalization chamber for 44, 53, and 61 days. Germination was staggered in such a way that all material would finish its vernalization on the same day. At this point, plants were transplanted to pots with two plants per pot, transferred to the greenhouse and grown under long day conditions (16 h day/8 h night). The number of days to the emergence of the first head was scored for each line. Both experiments were terminated after 100 days. The experiment was repeated twice. Minimal and the mean number of days to heading after the three periods of vernalization were used for calculation of QTLs. Lines that failed to head after 100 days were assigned “100 days to heading.” Distribution of the traits data was checked and the normal distribution hypothesis (Shapiro–Wilk test, *P* < 0.01) was not rejected. Broad-sense heritability (h^2^) estimate of vernalization requirements (in days) calculated from variance components (Czyczyło-Mysza et al. 2013) was 92%. Composite interval mapping analysis was performed using QTL Cartographer 2.5 software (Wang et al. 2012). After performing a 1000-permutation test, a LOD threshold was set individually to 2.9 to declare a QTL as significant. A walk speed of 1.0 cM was chosen for all QTL detections. QTL effects were estimated as the proportion of phenotypic variance (*R*^2^) explained by the QTL.

### In silico analysis

Sequences of wheat and rye DArT clones from QTLs were used to identify homologous or syntenic scaffolds in wheat genome. The scaffolds were identified using Basic Local Alignment Search Tool (BLAST) for wheat sequences stored in EnsemblPlants (http://plants.ensembl.org, release 36). Protein or nucleotide sequences corresponding to genes located in scaffolds were subsequently characterized for homologs using BLASTP or BLASTX in NCBI database (https://blast.ncbi.nlm.nih.gov/, update 24 July, 2017, 130,076,561 sequences), and putative functions were identified at UniProtKB database (http://www.uniprot.org/uniprot/). Additionally, released and annotated rye draft genome at the IPK Rye Blast Server was searched for selected rye DArT clones. Respective contigs were found, used for identification of transcripts (Bauer et al. 2017), and functionally annotated using Blast2GO (Conesa et al. 2005). The sequences of triticale DArT markers were recently released (Gawroński et al. 2016) and are accessible from National Center for Biotechnology Information.

## Results

### Doubled haploid line production

Line development was subdivided among all participants with parts of some populations created in more than one laboratory. Because of local adaptations of the techniques and major differences in experience, no direct comparisons of efficiencies can be made, and this was never the intended goal of the exercise. Overall, anthers from about 2300 heads were plated and over 2000 green plants were recovered (Table 2). Cv. Krakowiak appeared to be the most recalcitrant while cvs. Mungis and Presto are the most amenable to androgenesis. Some proportion of green plants were aneuploids, different in different populations (Oleszczuk et al. 2011), ranging from several percent to as high as 78% in population NEST, which in the end was never developed into a mapping population because the frequency of euploid plants was too low. All reasonably viable plants were colchicine treated to double their chromosome number. Complete seed set data were collected only for one treatment, in the MUST population where among 254 treated plants, 225 set seed. Among them, 41 were completely fertile, presumably as a consequence of spontaneous doubling of the chromosome number. If this criterion is correct, spontaneous doubling of the chromosome number took place in 18.9% of viable and vigorous androgenic regenerants observed here; the actual rate might have been higher but it would be confounded by aneuploidy. Some aneuploids were completely sterile even with their chromosome numbers doubled. The rate of chromosome doubling by colchicine, and associated with it the survival rate of treated plants, varied considerably among participating laboratories, with up to 95% of treated plants dying in one attempt to 100% survival in another. Again, these proportions are confounded by aneuploidy.

**Table 2:** The efficiency of androgenic progeny production in the tested F_1_ hybrids

Combination	Number of heads	Green plants recovered	Ratio, green plants per head
NECT	320	362	1.13
NEST	107	112	1.05
MUST	205	415	2.02
MUKR	179	211	1.18
MUCT	491	428	0.87
KRCT	639	234	0.37
KRST	346	286	0.83
Total	2287	2012	0.88

The target population sizes for each hybrid were ca. 250 DH lines, but this was achieved only in two combinations. Some F_1_ hybrids were recalcitrant, and despite repeated attempts, often by different participants, fewer progeny lines could be developed. Of two combinations involving NE422T, only that with Presto (NECT) produced a good number of green plants, but the effective size of the DH populations was reduced to 97 lines by a high frequency of aneuploids and high mortality after colchicine treatment (Oleszczuk et al. 2011). Hybrid NEST was as amenable to androgenesis as NECT, but almost all recovered progeny were aneuploids and only eight euploid DH lines were obtained. The effective sizes of some populations were further reduced by the presence of clones, which were detected after genotyping (Oleszczuk et al. 2014). After removal of clones and aneuploids, the set mapping populations consist of the following number of DH lines: 144 lines in MUKR, 172 lines in MUST, 138 lines in KRST, 231 lines in MUCT, of which 146 originate from a cross of Presto RM1B × Mungis and 85 from Presto × Mungis; 175 in KRCT, of which 72 are from the cross Presto RM1B × Krakowiak and 103 from Presto × Krakowiak; and 97 in NECT, for the total of 957 lines (Table 1).

### Genetic diversity analysis

The six mapping populations were derived from crosses involving five parental lines. Crosses of Mungis, Krakowiak, “Stan,” and “Presto” were in incomplete diallel design while “NE422T” was used in a single cross (Table 1). Cluster and principal coordinate (PCA) analyses based on the Dice similarity coefficients computed for 1154 loci showed that genetic diversity was not equally distributed among the components. Parental lines can be divided into two groups, the first composed of NE422T and Mungis and the other one gathering Presto, Krakowiak, and Stan 1 (Online Resource 7). Genetic relationships between the populations studied were measured directly as number of shared bands and Dice similarities (Online resource 8). Genetically, the most distant populations were NECT vs. KRCT and KRCT vs MUST with lowest values of similarities, and the last relationship was also evident by the number of shared bands.

### Linkage maps of biparental populations

As indicated above, the effective sizes of mapping populations were reduced by the presence of clones. Clone frequencies ranged from 6.7% in NECT to 34% in MUCT and MUKR populations. The effective numbers of genotypes ranged from 231 (MUCT) to 97 (NECT). The average number of DArT markers polymorphic in populations was 1337, and it ranged from 996 in KRST to 2041 in NECT (Table 1). For all but one mapping population, linkage groups corresponding to 21 chromosomes were identified (Table 3). Markers located on chromosome 7R were not detected in population KRCT. In 34 cases, 2 linkage groups (that could not be linked at LOD equal to 3.0) represented a single chromosome. The most of such split linkage groups were found for the genome A (23) as compared to B and R genomes with 6 and 5 chromosomes represented with double linkage groups, respectively. The number of double linkage groups corresponding to single chromosomes varied from 9 to 4 in MUKR and KRST populations, respectively (Table 3).

**Table 3:** Number of markers (unique and total) and length of biparental and consensus maps. Values separated by slash correspond to double linkage groups identified for a given chromosome

	1-MUKR			2-MUST			3-MUCT			4-KRST			5-KRCT			6-NECT			CONS		
	Unique	Total	cM	Unique	Total	cM	Unique	Total	cM	Unique	Total	cM	Unique	Total	cM	Unique	Total	cM	Unique	Total	cM
1A	7/3	9/16	31.5/5.6	15	32	104.7	9/5	26/19	3.9/50.6	11	15	115.6	3/2	5/4	8.7/6.8	20	40	143.6	35	82	154.1
2A	4/7	5/9	9.4/41.9	5/7	5/12	6.6/66.6	5/3	9/4	4.8/6.5	5/11	6/19	13.7/73.3	12	15	75.3	18/3	42/3	155.1/34.4	39/3	66/3	156.3/34.4
3A	17	27	120.2	11/6	14/7	47.8/24.3	20	39	128.9	6/6	10/8	41.2/20.5	8/13	9/17	21.7/48.3	28	49	172.7	58	85	183.6
4A	17	37	101.3	7	10	25.6	4/6	4/39	15.6/9.9	17	34	87.6	5	5	65.9	22	69	158.1	51	103	240.6
5A	7/4	8/5	24.3/34.4	5/4	7/6	41.5/21.6	12	16	93.5	8	11	59	6/5	6/6	48.4/39.9	5/4	9/4	37.6/9.6	27	35	177.1
6A	17/4	25/9	55.3/6.5	17/7	30/9	48.4/8.5	8/6	15/9	57.3/11.8	10	15	42.5	15	20	75.5	17	48	86.4	42/13	70/17	115.6/19.2
7A	22/3	32/3	89.1/7.3	20/6	28/7	100.2/21.6	9/4	14/4	16.9/1.8	9/7	14/7	31.5/56.5	20	32	109.9	11/6	20/13	37.9/28.8	61	86	219.2
Genome A	112	185	526.8	110	167	517.4	91	198	401.5	90	139	541.4	89	119	500.4	134	297	864.2	329	547	1300.1
1B	23	46	136.1	29	38	118.8	21	58	92.1	26	54	130.8	21	38	108.8	27	62	146.5	71	120	250.6
2B	23	29	177.6	28	55	128.9	29	55	120.3	24	44	117.6	37	59	179.6	27	77	162.9	84	139	253.6
3B	21	43	129.5	31	55	155.4	17	6	91.6	18	38	87	16/3	24/18	105.1/6.4	22/5	36/6	108.6/26.8	66/5	115/6	222.1/26.8
4B	4/1	6/2	35.5/0.0	6	7	66.8	3	7	6	6	6	57.8	11	14	97.3	8	15	72.8	21	29	96.7
5B	17/2	26/2	103.4/1.2	16	22	114.5	15	33	102.3	16	19	110.1	5/15	8/26	31.6/91.1	22	54	132	49	88	169.6
6B	24	51	134.9	25	57	102.6	13	48	66.5	14	17	120.8	35	90	208.1	21/10	78/20	69.7/38.9	76	153	257.8
7B	18	36	117.2	12	16	79.4	6	23	13.8	19	40	105.7	17	28	122.1	24	58	150.6	62	108	206.5
Genome B	133	241	835.4	147	250	766.4	104	230	492.6	123	218	729.8	160	305	950.1	166	406	908.8	434	758	1483.7
1R	43	88	112.8	31	106	80.5	12	47	85.3	35	91	99.7	43	121	172.3	28	61	121.4	98	228	260.9
2R	29	55	119.8	39	85	88.4	12	37	26.7	32	72	127.1	23	30	54.1	11/6	22/15	40.1/17.9	93	166	208.4
3R	3/24	6/53	6.1/80.6	30	82	74.4	4	5	3	31	88	63.2	6/28	8/53	21.7/88.1	43	113	124.4	94	184	184.3
4R	55	163	168.7	62	161	158.2	56	184	108	30	61	133.3	69	144	204.9	54	248	159.7	172	400	327.5
5R	4/24	10/57	7.9/59.3	31	98	129.6	26	83	68.9	18/20	38/44	75.3/47.0	23	54	82.9	40	124	164.5	117	263	264.7
6R	43	135	141	37	78	114.1	47	145	111.7	42	76	123.9	51	87	165.3	49	123	193.2	147	308	308.6
7R	33	99	90.8	40	108	133.4	29	101	59	45	124	95.1	–	–	–	30	150	81.4	92	229	206.9
Genome R	258	666	787	270	718	778.6	186	602	462.6	253	594	764.6	243	497	789.3	261	856	902.6	813	1778	1761.3
Total	503	1092	2149.2	527	1135	2062.4	381	1030	1356.7	466	951	2035.8	492	921	2239.8	561	1559	2675.6	1576	3083	4545.1

On the average, individual biparental maps were composed of 491 unique DArT markers with these ranging from 382 in MUCT to 572 in NECT (Table 3 and Online resources 1–6). The total number of polymorphic markers ranged from 921 (KRCT) to 1559 (NECT), but usually, over one half of them (from 63% for NECT to 46% in KRCT) were redundant. The average marker saturation calculated for unique DArT markers in individual genetic maps was 4.2 (cM/DArT marker), and it ranged from 3.6 (MUCT) to 4.6 (NECT). Numbers of redundant markers were lower for wheat genomes (43.3 and 49.6% for genomes A and B, respectively) and the highest for the rye genome (62.2%). Genome A had the lowest coverage with DArT markers with an average of 104 markers mapped in individual populations. The highest marker number per genome was found for genome R (248 markers), what resulted in a better saturation of the R genome in individual populations (3.0) when compared to genomes A and B (5.3 and 5.6, respectively).

### The consensus map of triticale

A consensus linkage map based on DArT markers was created from the six biparental maps by merging information on marker positions in individual maps. The consensus map consists of 3086 markers, of which 1576 are unique, and covers the distance of 4593.9 cM with the average map density of 2.9 cM (Table 3). Making use of three parents in three partially diallel crosses improved marker coverage of the consensus map with common markers (63.6%). The highest was the share of markers between populations MUKR vs MUCT and MUCT vs NECT (523 and 489, respectively). Between populations MUST and KRCT 256, simultaneously common markers were present (Table 3). In the consensus map, four markers showed three different locations in different biparental populations: two markers (rPt-506976.1-3, rPt-507094.1-3) on chromosomes 1R, 4R, and 5R and two markers (wPt-2632.1-3, tPt-3786.1-3) on chromosomes 4A, 6A, and 6B. The 104 markers mapped to two different loci on individual linkage maps (Online resource 9). The 41 and 22 of such double locations were within the rye and wheat genomes, respectively. Generally, markers with multiple locations were randomly dispersed across the genomes; however, in some cases, sets of such markers were identified. A cluster of nine markers spanning ~ 10 cM located distally on chromosome 6B in KRCT corresponds to the separate linkage group 6A.2 in the three populations with Mungis. Cluster of 7 rye markers were mapped on 7B of NECT population and 7R of MUST. The eight markers with double positions clustered on 1R chromosome of KRCT and three of them (rPt-399397, rPt-505577, rPt-505973) were mapped on 5R of MUST and KRST populations, while the remaining five markers (rPt-389501, rPt-389670, rPt-400535, rPt-400883, rPt-402101) were found on 4R chromosome of NECT.

Three chromosomes of the consensus map were composed of two separate linkage groups. In chromosomes 2A and 3B, groups of unique markers were derived from population NECT and had no common markers with the remaining clusters, and so they remain separated. Two separate linkage groups corresponded to chromosome 6A in three populations involving Mungis. In total, 329, 434, and 816 loci were mapped on genomes A, B, and R. This result corresponds to increasing mean number of unique markers on chromosomes from these genomes and decreasing average marker density (3.9, 3.4, and 2.2, for A, B, and R genomes, Table 3 and Online resource 10).

As a quality check, orders of loci in each chromosome of individual genetic maps were compared with respective positions on chromosomes of the consensus map (Online resource 11). Fractionated chromosomes are visible as new linear clusters of points starting from the middle or the end of the consensus map axis. In most cases, the consensus map presented a good compromise for individual maps. Despite fragmented linkage groups for 14 chromosomes, sufficient marker overlap permitted consensus, creating continuous linkage groups for all but three chromosomes (2A, 6A, and 3B). Minor disturbances in collinearity were found due to (a) suppressed recombination for fragments of chromosomes (2A, 4A, 7B in MUCT, 3B in KRCT), (b) rearrangements of chromosome fragments (2A size 20 cM in KRCT), and (c) transposition of clusters of 2–3 markers corresponding to single loci (7A, 3B, and 3R in KRCT, 5R in KRST).

### Segregation distortion

Markers showing distorted segregation were not eliminated during map construction; in biparental populations, they accounted on an average for 28.8% of unique markers, ranging from 21.4% in KRST to 34% in MUCT and KRCT. Three types of distortion can be discerned based on the frequencies of loci with alleles preferring maternal or paternal type. In the neutral type, similar number of loci with distorted alleles preferring mother or father pattern can be observed, as in MUKR (65 and 69 loci with allele preference to Mungis and Krakowiak, respectively) and NECT (67 and 63 loci with allele preference to NE422T and Presto, respectively). Preference for transmission of maternal alleles was observed in populations KRST (69 loci of the maternal type vs. 31 loci of the paternal type) and KRCT (117 to 52 loci biased towards Krakowiak and Presto, respectively). In the third type, distorted alleles more frequently inherited from the pollinator were found in MUST (66 and 113 loci preferring maternal and paternal type, respectively) and MUCT (56 and 75 loci preferring the maternal vs. the paternal type, respectively) populations.

Segregation distorted regions (SDRs) were identified based on five consecutive markers with distorted segregation, what corresponds to 1% of average number of non-redundant markers in the six mapping populations (mean of 491 markers) (Online resource 12). Markers with distorted segregation were mapped predominantly on chromosomes from the rye genome, although in MUST, most chromosomes of the B genome also contained SDRs. For the rye genome, there appeared no rule determining preferences for chromosomes with marker distortion but chromosomes 5R and 7R appeared to be involved more often than the remaining five rye chromosomes.

### Recombination of RM1B chromosome

Chromosome RM1B was introduced in DH lines derived from hybrids with “Presto RM1B.” In the three populations MUCT, KRCT, and NECT, a number of unique markers mapped to 1R chromosome amounted to 12, 43, and 28, respectively. Populations MUCT and KRCT were tested for the presence of storage proteins (*Gli-B1*, *Sec-1*, and *Glu-D1*), and lines with chromosomes 1R, 1RM1B, and recombinants were identified. PCA analysis of Manhattan distances for segregations of unique loci mapped to 1R showed no subpopulations corresponding to progeny obtained with Presto-RM1B, and Presto in MUCT and KRCT populations (Online resource 13a,b). However, within a subpopulation of MUCT (Online resource 13c), genotypes were divided into a group carrying 1R and the second group carrying RM1B. In a subpopulation of KRCT, lines with RM1B chromosome were separated from the remaining genotypes (Online resource 13d).

**Fig. 1:**
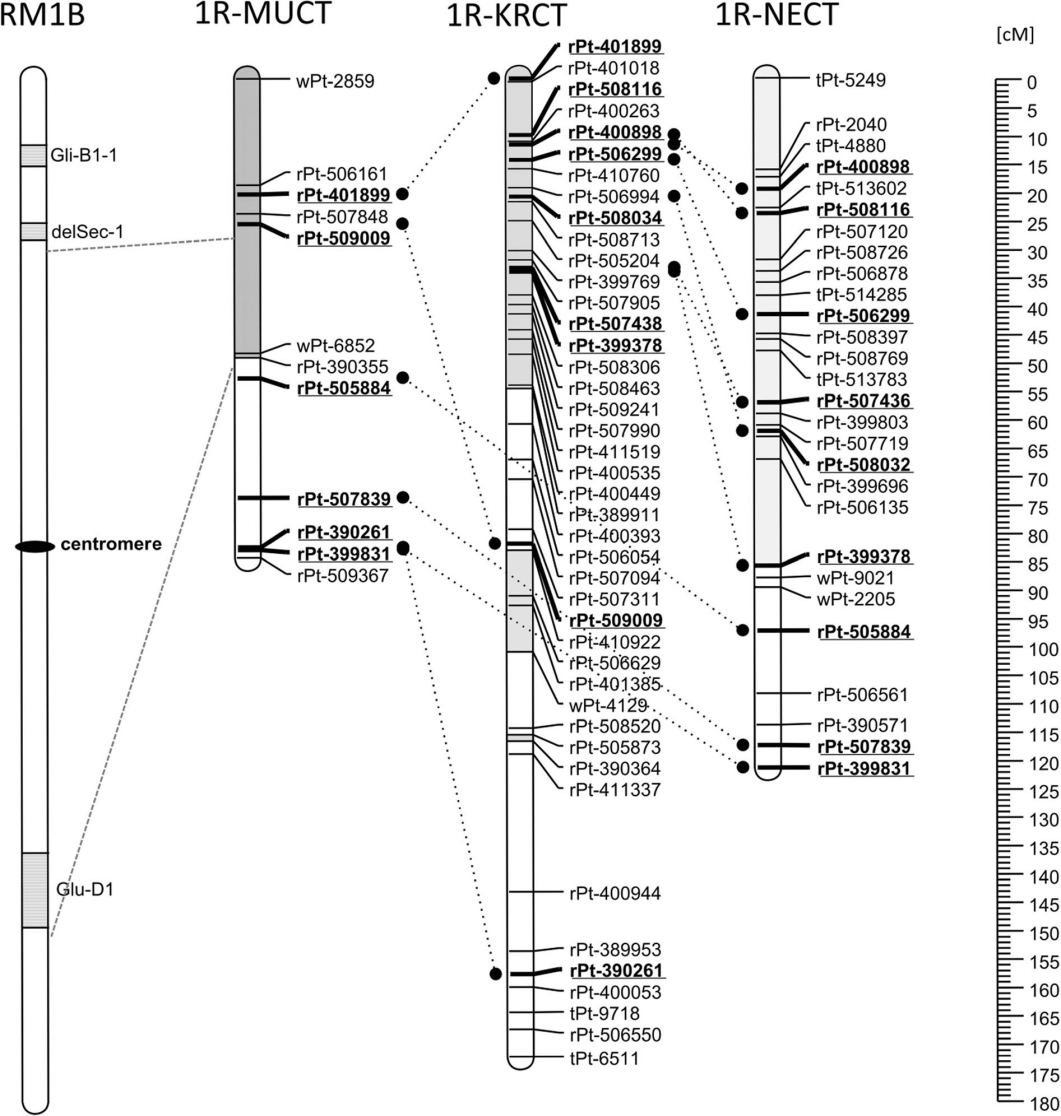
Chromosome RM1B with three segments originating from wheat (marked with dashes) from wheat (Lukaszewski 2006) and genetic linkage groups corresponding to 1R chromosomes in populations MUCT, KRCT, and NECT. Blocks of markers present in lines carrying translocated chromosome RM1B found in MUCT and KRCT were used to predict corresponding blocks of markers in NECT population. Homologous loci are underlined and bold and connected with dotted lines

In a subpopulation of MUCT, DH lines with intact RM1B (no recombination, all three wheat segments present) are expected to carry solely paternal alleles, and 20 out of 44 lines typed with PAGE as carrying RM1B followed this pattern. Another 20 RM1B PAGE-predicted lines carried evidence of recombination outside of the block of 7 markers marked with a shadowed bar (Fig. 1, 1R-MUCT). Therefore, the region of 1R chromosome with the complete of the three wheat segments corresponds to the discerned part of 1R MUCT chromosome. The pattern of 7 paternal alleles was found in 40 lines (91%) with RM1B predicted by PAGE analysis. Moreover, for 19 lines, recombination between the *Glu-D1* and *Gli-B1* loci was demonstrated by PAGE and this corresponds to the breakpoint between markers rPt-509009 and wPt-6852 present in 17 of the recombined RM1B lines. Thus, the region of loci wPt-6852 and rPt-390355 is associated with *Glu-D1*.

A subpopulation of 72 KRCT DH lines was obtained from crosses with Presto RM1B. PAGE has shown that 18 of these 72 lines carry RM1B and 3 lines showed evidence of recombination between the *Glu-D1* and *Gli-B1* loci. The region composed of 23 markers (flanked by rPt-401899 and rPt-389911), and a set of two markers (rpt-505873 and rpt-390364) was present in 16 and 18 RM1B lines, respectively. Taking into account the collinearity of maps, the region corresponding to the *Glu-D1* locus in KRCT subpopulation should be expected below marker rPt-509009. Within the RM1B carrying lines, no line was composed exclusively of paternal alleles, and in all these lines, group of 5 loci flanked by markers rPt-509009 and wPt-4129 was of maternal type.

Comparative maps of RM1B chromosomes from MUCT and KRCT allows proposing the set of 20 unique DArT markers (from tPt-5249 to rPt-506135) as associated with the presence of RM1B chromosome in the NECT population. Fifteen NECT lines with this set of paternal allele were identified (NECT_1001, NECT_1002, NECT_1014, NECT_1017, NECT_1018, NECT_1021, NECT_1023, NECT_1030, NECT_1041, NECT_1049, NECT_1051, NECT_1055, NECT_1056, NECT_1096, and NECT_1062).

### QTL analysis of the vernalization requirement

An analysis of the minimal vernalization requirement triggering the generative stage and the average number of days to heading revealed two QLTs on chromosomes 7B and 2R (Table 4). These QTLs explained 5.6–8.2% of variation. Sequence analysis was performed to identify candidate genes in the identified regions. Sequence data for clone wPt-3873 was not available so two adjacent DArT markers with available sequences (wPt-6372, co-localized, and wPt-6498, + 0.52 cM upstream) were selected based on wheat map of Huang et al. (2012). Sequences of wPt-6372 and wPt-6498 were blasted in EnsemblPlants database (http://plants.ensembl.org), and respective scaffolds 578086 (7BL:6343–6703) and 579015 (7BL:1516–2035) were identified. In the first scaffold, three genes were found but the corresponding proteins (UniProt: A0A1D6SDL6, A0A1D6SDL7, and A0A1D6SDL8) were not related with vernalization requirements. The second scaffold contained a single gene (UniProt: A0A1D6CAE5) coding an ALP1-like protein that is an element of the POLYCOMB REPRESSIVE COMPLEX2. ALP1 protein is needed for full reactivation of several floral homeotic genes that are repressed by genes of the Polycomb group (Liang et al. 2015). Analysis of genes predicted based on homology in locus QVrn.2R revealed 15 genes (Online materials 14). Two of them (transcription initiation factor TFIID subunit 9-like and AP2/ERF and B3 domain-containing protein) are involved in the regulation of gene expression. However, more evidence is needed to connect the action of these genes with the regulation of the vernalization process. DArT sequences from region of QVrn.2R were mapped to nine contigs (60,155, 292,872, 76,764, 59,900, 2929, 1,354,753, 289,064, 127,588, 2,872,817) of Lo7 rye draft genome (Bauer et al. 2017). In total, five genes were annotated functionally in the selected contigs (citron Rho-interacting kinase-like, calcium-dependent kinase 7, 3-isopropylmalate dehydratase large subunit, guanosine-3,5-bis(diphosphate) 3-pyrophosphohydrolase, and transcription factor RF2b-like isoform X1). According to GO annotation, these transcripts were not directly involved in vernalization process. This should be also underlined that phenotyping in multiple environments is necessary to precisely localize QTLs (Melchinger et al. 1998).

**Table 4:** QTLs of vernalization requirements in KRST population measured as number of days to heading (DTH) after minimal vernalization time (MinDTH) sufficient for meristem transition from vegetative to generative stage and average (AvDTH) for three vernalization periods (44, 53, and 61 days)

Locus	Region (cM)	Trait	Flanking markers	LOD	R^2^ [%]	Additive effect	Additional markers
QVrn.7B	0–1.7	MinDTH	wPt-3873	2.24	6.4	16.4	wpt-6372
	0–3.1	AvDTH	wPt-3873	2.36	6.8	17.3	
QVrn.2R	57.6–59.8	MinDTH	rPt-507782, rPt-398678	2.02	5.6	15.9	rPt-509138, rpt-402364
	61.7–59.0	AvDTH	rPt-506855, rPt-399333	2.60	8.2	19.5	rpt-506926, tpt-513861

## Discussion

Hexaploid triticale is a relatively new human-made crop that slowly but consistently grows in acreage in many areas of the world. As a new crop, it offers unique opportunities to study evolution of allopolyploids and the effects of selection for agronomic performance. Even cursory observation of triticale over its brief history clearly illustrates interactions among constituent genomes and their responses to selection pressure. Early triticales suffered from considerable meiotic instability and hence high aneuploid rate, kernel shriveling, and pre-harvest sprouting. Strong breeders’ selection reduced these problems to some extent but did not eliminate them completely. While present day commercial triticale cultivars are meiotically quite stable, occasionally, aneuploidy creates registration problems as aneuploids deviate morphologically from euploids (Oleszczuk and Banaszak 2016). Poor chromosome pairing is quite common in hybrids. Here, poor pairing and resulting aneuploidy were a problem in the creation of the current set of lines, with NEST being completely rejected. Similarly, kernel shriveling is no longer a major problem in breeding as most advanced breeding materials have acceptable grain volume weights. Pre-harvest sprouting remains a serious problem, only partially inherited from rye. All these characteristics, as well as many others, are represented among the parents chosen for the creation of the set of mapping populations and can be studied in detail. Crosses were designed in such a way so that each parent in present in at least two combination; unfortunately, NE422T × Stan 1 was not only recalcitrant to androgenesis but its extreme aneuploid rate (93%) made the task of developing a full mapping population impossible. The authors believe that by using each parent in at least two cross combination and instant verification of mapping effort can be obtained for characteristics differentiating the parental lines.

At present, triticale is not viewed as a bread-making crop. However, good-quality bread can be baked with somewhat modified technology; for strictly wheat-like bread-making technology, a set of engineered chromosomes was created that restore in triticale almost wheat-like gluten composition (Lukaszewski, 2006). One of these chromosomes, RM1B, was incorporated in portions of two mapping populations, its presence detected electrophoretically and a set of identifying DNA markers selected. At the distal end of 1RS, the MUCT population shows a cluster of markers wPt-11939.1, wPt-2859.1, wPt-7905.1, and wPt-8261.1 that also to map to the region of 48–62 cM on the consensus of wheat chromosome 1B. These markers likely represent two small introgressions from 1BS into 1RS that introduce *Gli-B1/Glu-B3* and eliminate *Sec-1*. DNA sequence of wPt-8261 was available (http://www.diversityarrays.com/dart-map-sequences) and showed 77% identity to prolamin gene locus of *Aegilops tauschii* (JX299977.2). Also, in the MUCT population, a wheat-derived marker normally mapping to the distal end of 1DL (wPt-4180, Marone et al. 2013) appears on chromosome 1RL. This marker very likely represents a 1DL segment replacing the *Sec-*3-carrying segment of 1R with a corresponding segment of 1D with *Glu-D1*.

An additional population of doubled haploids from Presto FC2 × Mungis has been developed but not genotyped and hence not included in the present set. Chromosome FC2 is an engineered 1R with all storage protein loci from wheat chromosome 1D (Lukaszewski 2006); the population is available from AJL.

An interesting elongation of the vernalization requirement has been observed among winter triticales emerging from Polish breeding programs in the last ca. 20 years. Cvs. Presto and Krakowiak are good examples of this change (A. Lukaszewski, personal communication). The standard vernalization treatment of Presto as applied in Riverside CA is 42–45 days under short days and temperatures between 4 and 7 °C. After transfer to the greenhouse and grown under long days (16 h day/8 h night), Presto heads within 6 to 7 weeks, depending on ambient temperature. Cvs Mungis, Stan 1, and NE422T behave in a very similar manner. Krakowiak, and many more recent cultivars from Polish breeding programs, requires at least 56–60 days of vernalization under the same conditions, if they are to head within 7 weeks of removal to standard greenhouse conditions. A simple test performed on the KRST population suggests that it is not a different allele at known vernalization loci that is responsible for the extended vernalization requirement of Krakowiak but rather a different regulatory element located elsewhere in the genome. At this point, it is not clear if this change represents a reaction of triticale to strong selection pressure for winter survival or if perhaps the character was transferred from winter bread wheat.

The overall level of map saturation was somewhat disappointing. It might have been greater with more distant crosses, but the guiding principle here was making use of crosses commonly made in breeding programs. It is believed that in collaborative efforts and additional genotyping, maps will become more saturated and gaps closed. Also, the total length of maps (ca. 4600 cM) is about twice as long as expected based on chromosome pairing frequencies and cytologically predicted chiasma frequencies. Rye chromosomes 4R and 6R produced particularly long maps (Online resource 10), for unclear reasons. Map inflation is most likely generated by mis-scored markers (Cartwright et al. 2007). While a majority of so-called singletons were eliminated, any further manual editing of marker score would only introduce an additional error of unknown magnitude and direction. The highest percentage of missing values was found in populations MUKR, MUCT, and NECT (11.8, 10.5, and 10.2%, respectively). Number of missing data in populations KRCT, MUST, and KRST was below 10% (8.0, 5.6, and 5.0%, respectively). To verify robustness of mapping, distances were recalculated after removal of individuals with number of missing data exceeding 10% (ESM 1-6). Reduction of mapping populations resulted in elimination of individuals with unique recombination events, overall reduction of map lengths by 7%, and decreased number of unique markers (reduction by 17%).

Chromosome maps generated here show a generally good agreement with published maps of wheat, rye, and triticale, with notable exceptions. Inverted orientation or rye chromosomes 2R, 4R, and 5R was previously reported (Kalih et al. 2014, Hackauf et al. 2017) in genetic maps of rye and triticale (Miedaner et al. 2012, Alheit et al. 2011). Comparative analysis in respect to rye draft genome extends this observation also to other maps of rye and triticale (Online resource 15). Major inconsistencies relative to published maps are on chromosomes 1A, 5A, 6A, 2R, and 3R. The comparative analysis of genetic maps indicates that the chromosomes 5A and putatively 6A in the consensus map published by Alheit et al. (2011) are inverted relative to previously published genetic linkage map of wheat and triticale. Using the DArT map of rye as a reference (Bolibok-Brągoszewska et al. 2009), we found high agreement of the proposed order of loci (correlations 0.93 and 0.87, respectively, for chromosomes 2R and 3R, Online resource 15). Differences in order of loci between our maps relative to the consensus map of Alheit et al. (2011) may result from better marker saturation and more even distribution of markers along these two chromosomes perhaps because of our retention of markers with distorted segregation. As for chromosomes 2A, 3A, and 3B, low correlation coefficients with wheat and high with triticale may indicate that in triticale, these chromosomes have undergone substantial and stable rearrangements. Only repeated mapping efforts in various populations may confirm such chromosomal rearrangements. The order of loci on chromosome 6A is different in wheat and triticale and perhaps the level of variation in triticale in this linkage group is too high to establish a robust consensus.

Relative to the consensus triticale map of Alheit et al. (2011), our map shows an introgression of a cluster of markers from chromosome 2B into the 58–72 cM region of chromosome 2A. Similarly, a set of nine markers located on chromosome 6A by Alheit et al. (2011), consensus-6A 98.5–109.2 cM, in our KRCT population, was linked to chromosome 6B. These nine markers also showed alternative positions on chromosomes 4A and 6A. Putative rearrangements involving chromosomes 2A, 2B, and 6A in triticale have already been suggested (Tyrka et al. 2011) and may represent a form of adaptive genome rearrangements in an amphiploid. Inconsistencies in position of genetic markers may influence detection and localization of effects contributing to plant height and biomass yield in a triticale (Busemeyer et al. 2013, Alheit et al. 2014, Liu et al. 2014, Würschum et al. 2014a, b, Liu et al. 2017).

The authors of this article treat this joint effort as one of the first steps to genetic dissection of triticale, a new human-made crop. With additional efforts, some discrepancies between different genetic maps will likely be removed; additional mapping efforts will likely increase map saturation and lead to identification of genetic loci critical for specific agronomic traits of triticale, and perhaps intergenomic interactions affecting agronomic traits. Small seed samples of all materials described here are freely available from AJL; larger samples can be requested form Dr. Jamie Larsen, Lethbridge Research and Development, Agriculture and Agri-Food Canada, 5403–1 Ave. South, P.O. Box 3000, Lethbridge, AB T1J 4B1. All genotype information for all lines can be obtained from MT and AJL.

## Electronic supplementary material

ESM 1 (XLSX 2950 kb)

ESM 2 Fig. S1 Distribution of the parents of the mapping populations across two first principal coordinates (PC) extracted from Dice genetic similarities (GIF 29 kb)

High Resolution Image (TIFF 11 kb)

ESM 3 (DOCX 23 kb)

ESM 4 Fig. S2 Schematic illustration of the consensus genetic map for six populations of DH triticale lines. Horizontal lines within each chromosome represent unique markers. Chromosomes 2A, 6A and 3B are represented by two linkage groups not connected at LOD equal to 3.0. These chromosomes can be connected in very low LOD (2A: 0.9, 3B: 1.8, 6A: 0.4–0.9) and at large distance (2A: 88 cM, 3B: 54 cM, 6A: 50–86 cM) (GIF 57 kb)

High Resolution Image (TIFF 791 kb)

ESM 5 Fig. S3 Representation of agreement of markers from six biparental maps with positions from consensus map (GIF 1056 kb)

High Resolution Image (TIFF 421 kb)

ESM 6 (DOCX 23 kb)

ESM 7 Fig. S4a-d Distribution of the of the 224 (a) and 174 (b) DH lines representing total mapping populations MUCT and KRCT, respectively. Distribution of 146 (c) and 72 (d) genotypes from subpopulations MUCT, and KRCT obtained with Presto-RM1B, respectively. Two first principal coordinates (PC) were extracted from Manhattan distances. DH lines obtained with ‘normal’ Presto’ were marked with crosses (JPEG 257 kb)

ESM 8 (DOCX 29 kb)
