# Supplementary figures and images for: Populations of doubled haploids for genetic mapping in hexaploid winter triticale
Source: Mol Breed. 2018 Mar 30;38(4):46. doi: 10.1007/s11032-018-0804-3 (PMC5878199; doi:10.1007/s11032-018-0804-3)

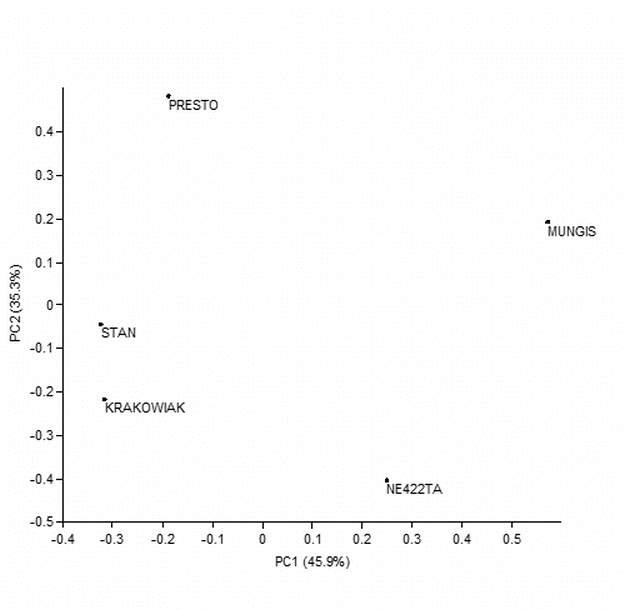

Supplement: Supplementary file 2 — Fig. S1 Distribution of the parents of the mapping populations across two first principal coordinates (PC) extracted from Dice genetic similarities (GIF 29 kb) [file 11032_2018_804_Fig2_ESM.gif]

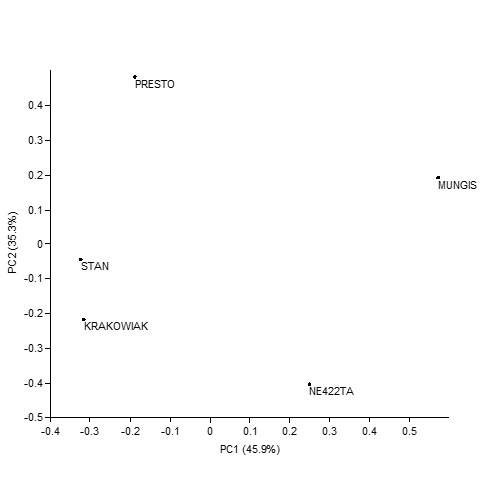

Supplement: Supplementary file 3 — High Resolution Image (TIFF 11 kb) [file 11032_2018_804_MOESM2_ESM.tif]

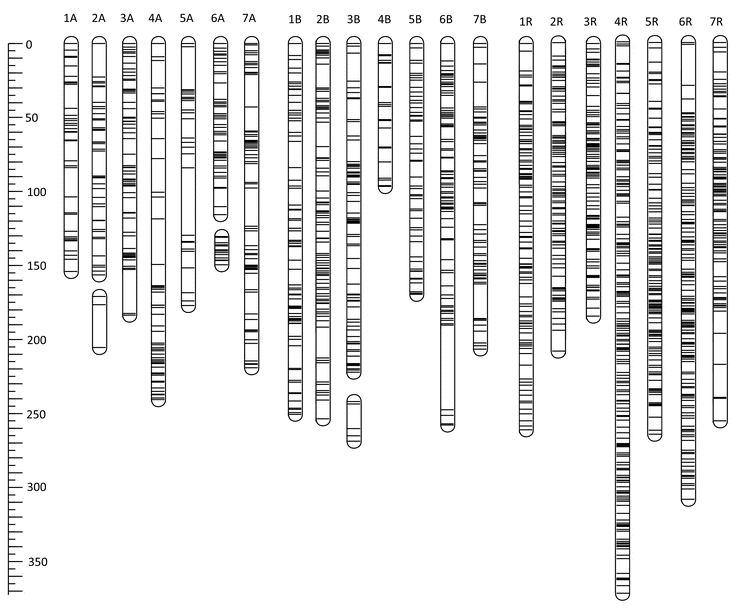

Supplement: Supplementary file 5 — Fig. S2 Schematic illustration of the consensus genetic map for six populations of DH triticale lines. Horizontal lines within each chromosome represent unique markers. Chromosomes 2A, 6A and 3B are represented by two linkage groups not connected at LOD equal to 3.0. These chromosomes can be connected in very low LOD (2A: 0.9, 3B: 1.8, 6A: 0.4–0.9) and at large distance (2A: 88 cM, 3B: 54 cM, 6A: 50–86 cM) (GIF 57 kb) [file 11032_2018_804_Fig3_ESM.gif]

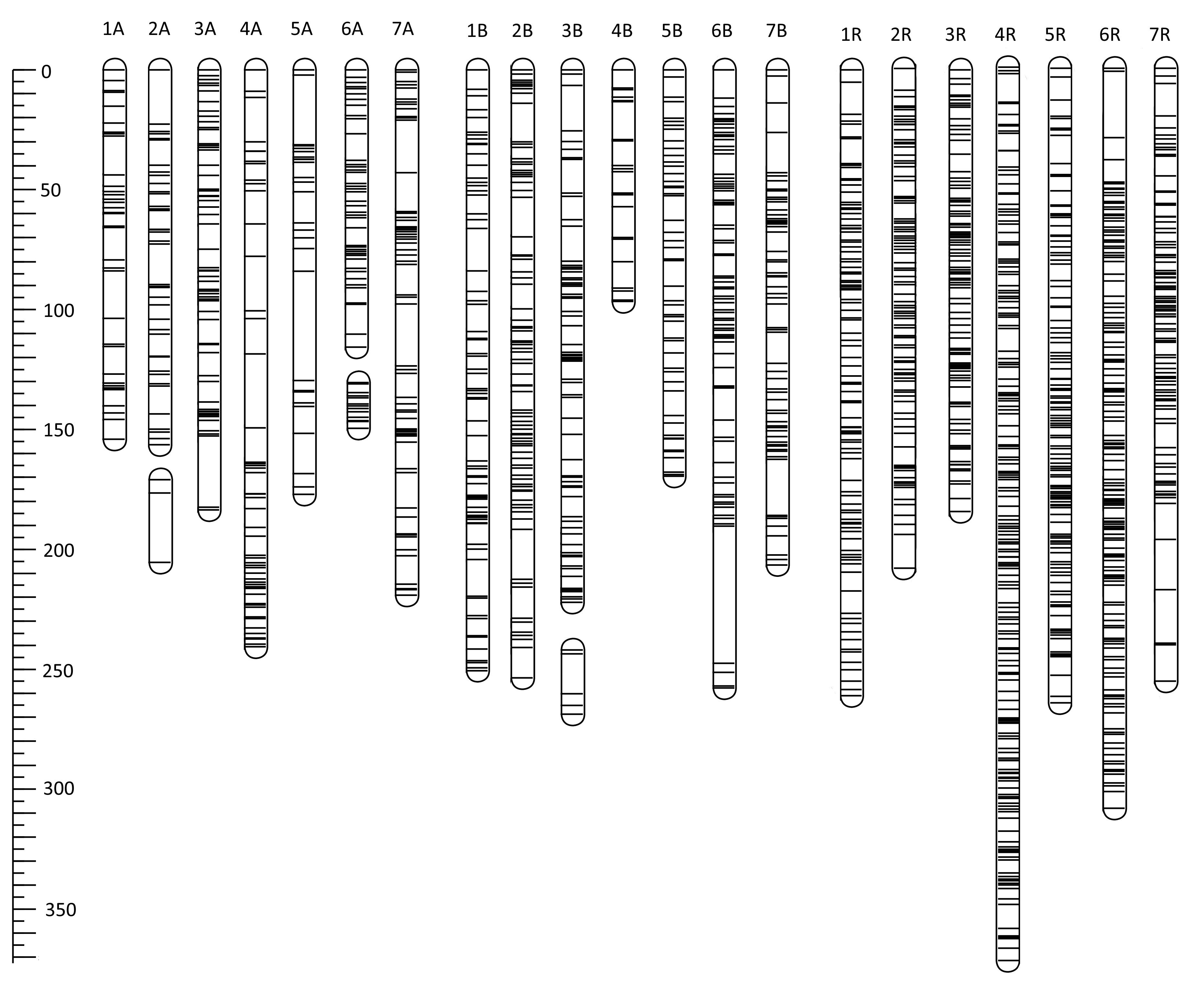

Supplement: Supplementary file 6 — High Resolution Image (TIFF 791 kb) [file 11032_2018_804_MOESM4_ESM.tif]

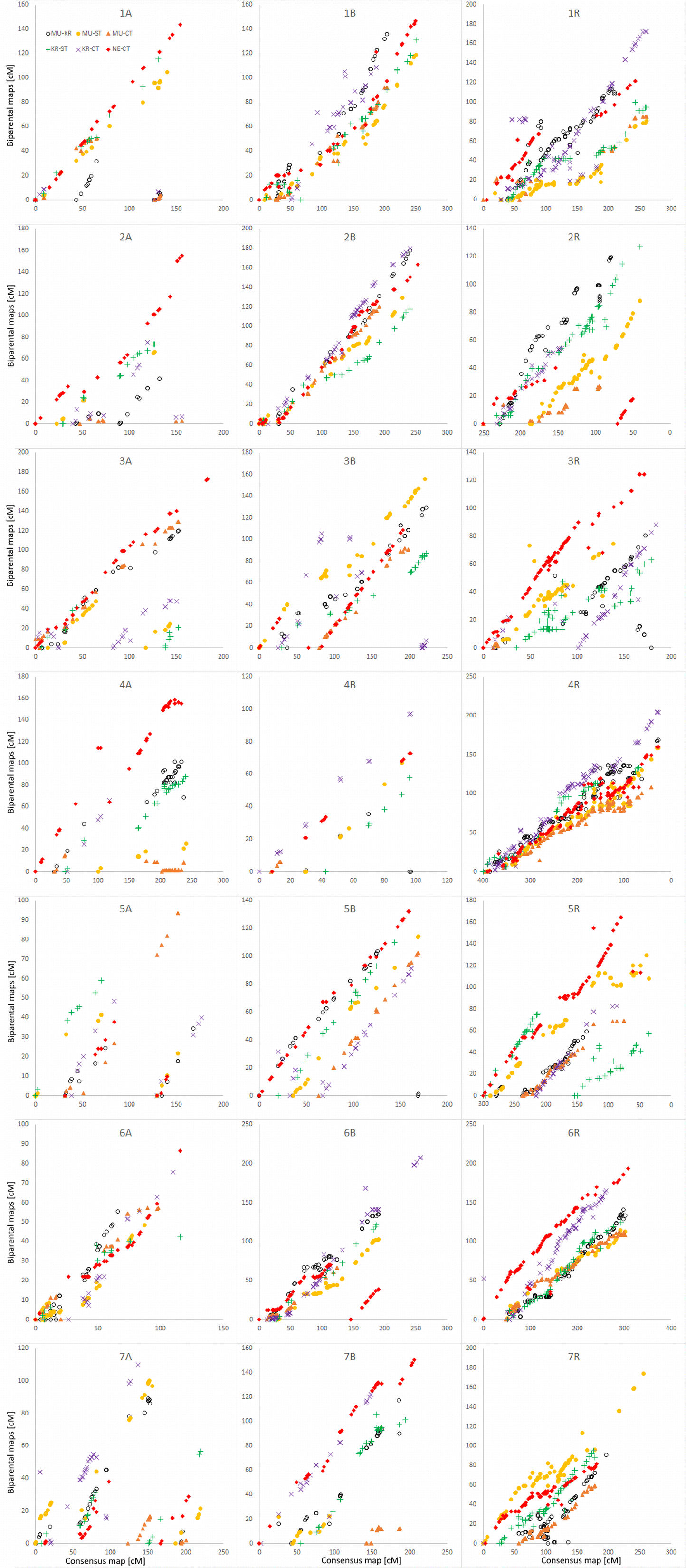

Supplement: Supplementary file 7 — Fig. S3 Representation of agreement of markers from six biparental maps with positions from consensus map (GIF 1056 kb) [file 11032_2018_804_Fig4_ESM.gif]

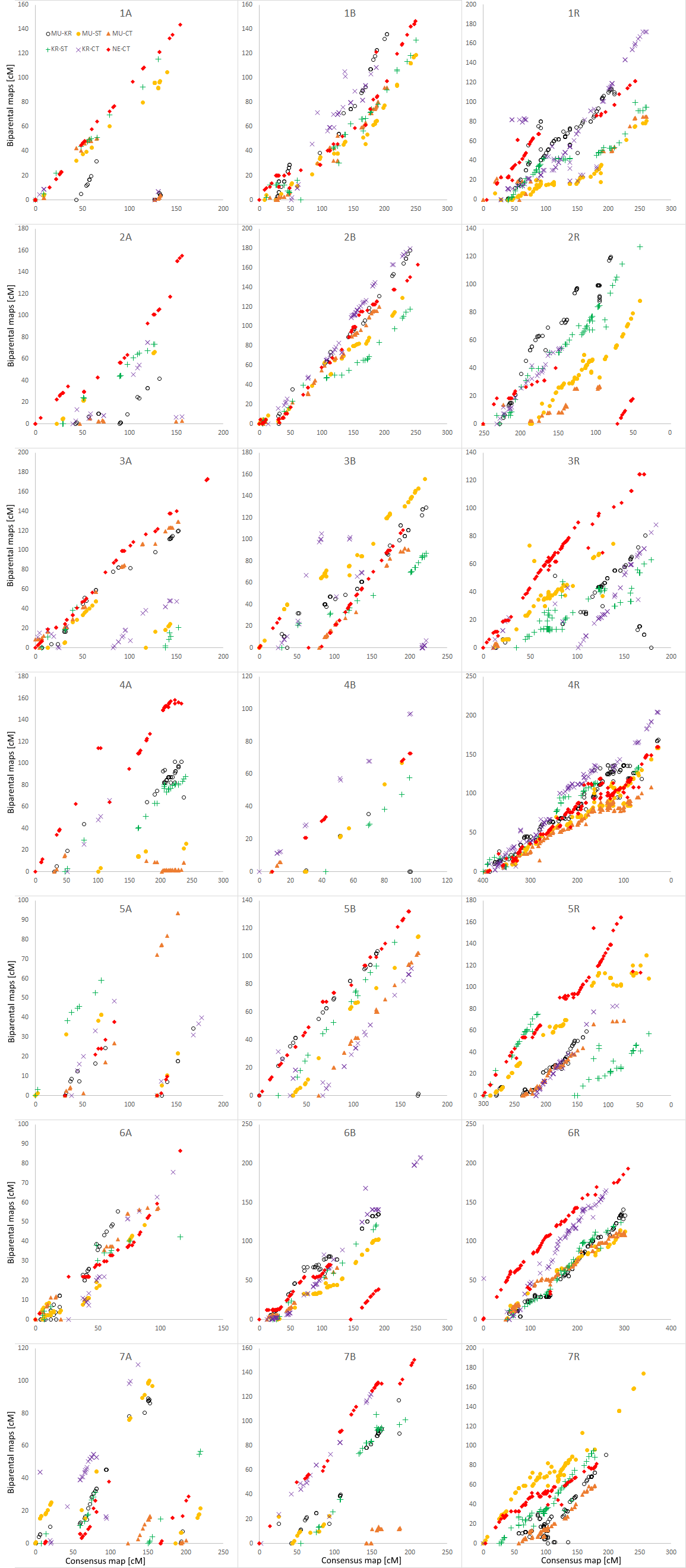

Supplement: Supplementary file 8 — High Resolution Image (TIFF 421 kb) [file 11032_2018_804_MOESM5_ESM.tif]

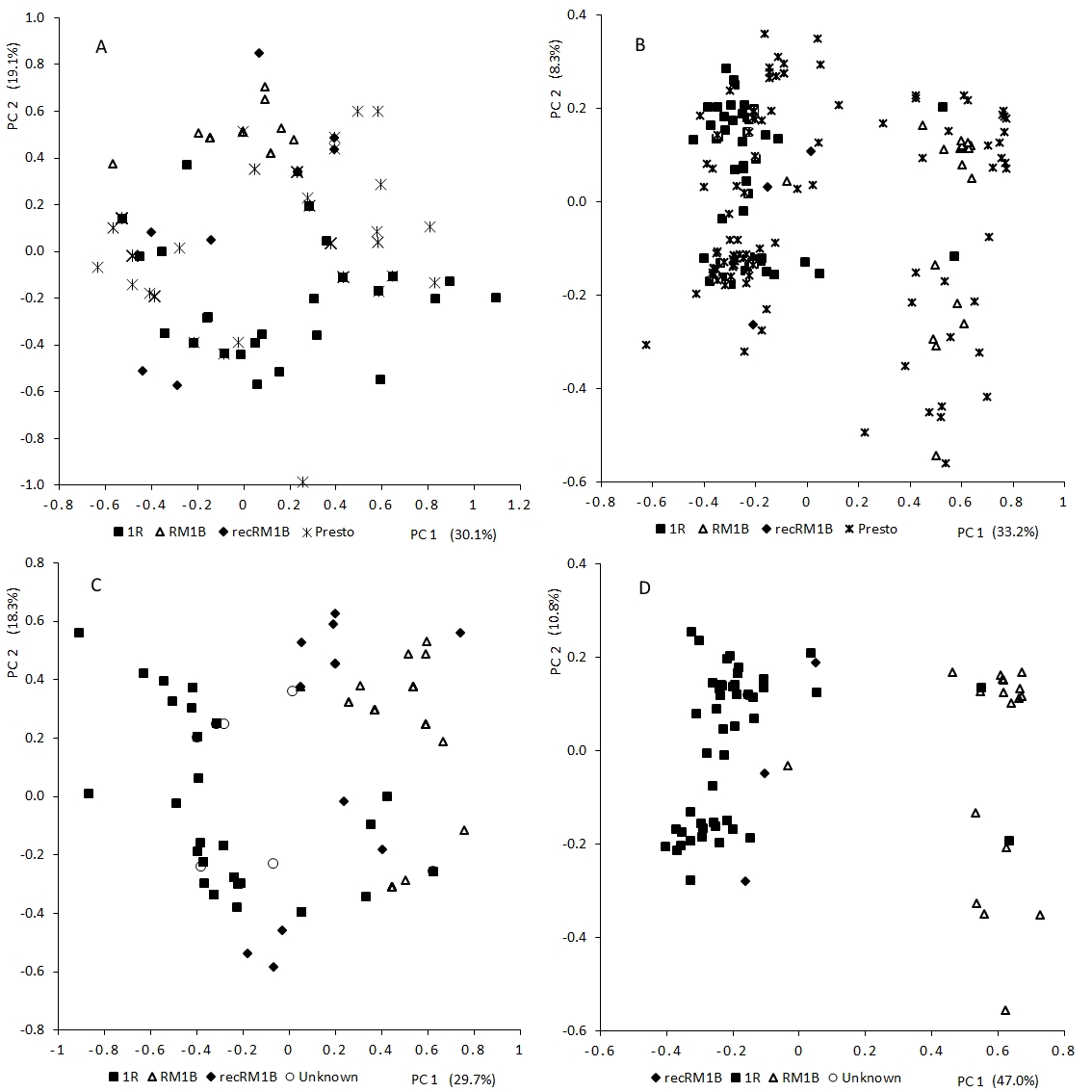

Supplement: Supplementary file 10 — Fig. S4a-d Distribution of the of the 224 (a) and 174 (b) DH lines representing total mapping populations MUCT and KRCT, respectively. Distribution of 146 (c) and 72 (d) genotypes from subpopulations MUCT, and KRCT obtained with Presto-RM1B, respectively. Two first principal coordinates (PC) were extracted from Manhattan distances. DH lines obtained with ‘normal’ Presto’ were marked with crosses (JPEG 257 kb) [file 11032_2018_804_MOESM7_ESM.jpg]
