# Supplementary material for: Populations of doubled haploids for genetic mapping in hexaploid winter triticale
Source: Mol Breed. 2018 Mar 30;38(4):46. doi: 10.1007/s11032-018-0804-3 (PMC5878199; doi:10.1007/s11032-018-0804-3)
Supplement: Supplementary file 4 — (DOCX 23 kb) [file 11032_2018_804_MOESM3_ESM.docx]

Table S8. Numbers of common markers (upper diagonal) extracted from the pool of 3083 markers located on the consensus map and Dice genetic similarities (lower diagonal) between mapping populations.

|  | MUKR | MUST | MUCT | KRST | KRCT | NECT |
| --- | --- | --- | --- | --- | --- | --- |
| MUKR | - | 418 | 523 | 417 | 413 | 444 |
| MUST | 0.375 | - | 359 | 402 | 256 | 457 |
| MUCT | 0.486 | 0.327 | - | 300 | 275 | 489 |
| KRST | 0.408 | 0.385 | 0.298 | - | 301 | 397 |
| KRCT | 0.412 | 0.250 | 0.278 | 0.322 | - | 304 |
| NECT | 0.335 | 0.339 | 0.373 | 0.316 | 0.246 | - |
