# Supplementary material for: Populations of doubled haploids for genetic mapping in hexaploid winter triticale
Source: Mol Breed. 2018 Mar 30;38(4):46. doi: 10.1007/s11032-018-0804-3 (PMC5878199; doi:10.1007/s11032-018-0804-3)
Supplement: Supplementary file 9 — (DOCX 23 kb) [file 11032_2018_804_MOESM6_ESM.docx]

Table S9. Distribution of SDRs across genomes in individual populations. Numbers of markers attributed to SDR in brackets

| Population | Genome A | Genome B | Genome R |
| --- | --- | --- | --- |
| MUKR | 1A(5), 6A(11), 7A(7) | 2B(5) | 4R(5,12,5), 7R(17) |
| MUST |  | 1B(11,7), 2B(17), 4B(5), 5B(8), 6B(8) | 2R(5,7), 3R(25), 5R(5), 7R(25) |
| MUCT | 5A(6), 7A(9) | 1B(5), 2B(14) | 5R(9,6,8), 6R(5,5), 7R(29) |
| KRST |  | 5B(8) | 2R(20), 4R(7), 5R(13) |
| KRCT | 3A(8), 7A(5) | 6B(9) | 1R(35), 4R(12,10,6,5) |
| NECT |  |  | 1R(23), 5R(21), 7R(5,21) |
