# Supplementary material for: Populations of doubled haploids for genetic mapping in hexaploid winter triticale
Source: Mol Breed. 2018 Mar 30;38(4):46. doi: 10.1007/s11032-018-0804-3 (PMC5878199; doi:10.1007/s11032-018-0804-3)
Supplement: Supplementary file 11 — (DOCX 29 kb) [file 11032_2018_804_MOESM8_ESM.docx]

Table S10. Genes identified in region of locus QVrn.2R. Function of corresponding wheat predicted genes (preliminary annotated as uncharacterized proteins) was established.

| Position on 2R chromosome [cM] | DArT markers | Scaffold TGACv1  (score, E-val, %id) | Predicted genes | Blastp/Blastx hits (NCBI ID; score, E-val, %id) |
| --- | --- | --- | --- | --- |
| 58.9 | rpt-506855,  rpt-507446,  rpt-506685 | 159996_2DL:35394-35525  (114, 1.0E-42, 93.2) | >tr\|W5C235\|W5C235_WHEAT | citron Rho-interacting kinase-like (XP_020170406.1; 814, 0.0, 91%) |
| 61.7 | rpt-506926 | 129550_2BL:141962-142117  (78, 1.3E-25, 75.0) | >tr\|W5B6J2\|W5B6J2_WHEAT | heme oxygenase 1, chloroplastic-like (XP_020155624.1, 525, 0.0, 96%) |
| 61.7 | tpt-513861,  rpt-506196 | 113210_2AS:47269-47602  (324, 5.8E-143, 98.5) | >tr\|A0A1D5TN00\|A0A1D5TN00_WHEAT | calcium-dependent protein kinase 20 (XP_020168962.1, 862, 0.0, 99%) |
| 62.7 | rpt-507782 | TGACv1_scaffold_146759_2BS:7511-7721 (193, 2.1E-80, 95.7) | >tr\|A0A1D5UB20\|A0A1D5UB20_WHEAT | 3-isopropylmalate dehydratase large subunit, chloroplastic (XP_020192783.1, 911, 0.0, 98%) |
| 64.3 | rpt-509138,  rpt-509132 | TGACv1_scaffold_147655_2BS:36486-36723 (234, 3.7E-100, 99.2) | >tr\|W5BNZ6\|W5BNZ6_WHEAT  >tr\|A0A1D5UDT4\|A0A1D5UDT4_WHEAT  >tr\|A0A1D5UDT5\|A0A1D5UDT5_WHEAT | 60S ribosomal protein L27a-3-like (XP_020185985.1;286, 8e-98, 97%)  probable GTP diphosphokinase RSH3, chloroplastic isoform X1 (XP_020185986.1; 1184, 0.0, 97%)  transcription initiation factor TFIID subunit 9-like (XP_020185989.1; 271, 2e-90, 90%) |
| 65.1 | rpt-398678 | TGACv1_scaffold_130673_2BL:38207-38451 (211, 3.4E-89, 93.1) | >tr\|A0A1D5U0Q4\|A0A1D5U0Q4_WHEAT | hypothetical protein TRIUR3_13773 (EMS46932.1; 244, 4e-81, 83%) |
| 65.1 | rpt-402364 | TGACv1_scaffold_178575_2DS:22565-22967 (353, 7.3E-157, 93.8) | >tr\|A0A1D5V0W6\|A0A1D5V0W6_WHEAT | transcription factor RF2b-like isoform X1 (XP_020158207.1, 749, 0.0, 100%) |
| 65.8 | rpt-399333,  rpt-402236,  rpt-410940,  rpt-410800 | TGACv1_scaffold_146686_2BS:89211-89320 (74, 3.2E-24, 83.6) | >tr\|A0A1D5UAS2\|A0A1D5UAS2_WHEAT  >TRIAE_CS42_2BS_TGACv1_146686_AA0470670.1  >TRIAE_CS42_2BS_TGACv1_146686_AA0470680.1  >TRIAE_CS42_2BS_TGACv1_146686_AA0470710.1  >TRIAE_CS42_2BS_TGACv1_146686_AA0470720.1 | Putative DNA repair and recombination protein RAD26-like protein (EMS60403.1, 296, 5e-93, 94%)  switch 2 (XP_020199702.1; 475, 1e-155, 96%)  AP2/ERF and B3 domain-containing protein Os05g0549800-like (XP_020183028.1; 228, 3e-72, 86%)  di-SUMO-like protein (ACL50300.1; 368, 2e-126, 92%)  putative reverse transcriptase (AAN05532.1; 435, 5e-137, 51%) |
| 65.8 | rpt-390663 | TGACv1_scaffold_149127_2BS:2830-2882 (, 51, 1.9E-13, 98.1) | >tr\|A0A1D5UFM6\|A0A1D5UFM6_WHEAT | uncharacterized protein LOC109781946 (XP_020196122.1, 580, 0.0, 89%) |

Table. S11. Correlation coefficients for the order of common loci (in brackets) between the consensus map generated here and linkage maps of rye (Bolibok-Brągoszewska et al. 2009, Bauer et al. 2017), wheat (Crossa et al. 2007), triticale consensus (Alheit et al. 2011) and biparental triticale ‘Saka3006’ x ‘Modus’ (SM, Tyrka et al. 2011)

| Chromosome | Rye | Draft rye genome | Wheat | Triticale consensus | Triticale SM |
| --- | --- | --- | --- | --- | --- |
| 1A | - | - | 0.719(23) | 0.368(24) | 0.985(10) |
| 2A | - | - | -0.229(10) | 0.752(37) | 0.962(20) |
| 3A | - | - | -0.005(17) | 0.969(50) | 0.983(20) |
| 4A | - | - | 0.877(18) | 0.900(50) | 0.959(10) |
| 5A | - | - | 0.999(3) | -0.483(21) | 0.990(9) |
| 6A | - | - | 0.129(24) | -0.031(64) | 0.328(32) |
| 7A | - | - | 0.724(13) | 0.943(54) | 0.866(18) |
| 1B | - | - | 0.826(20) | 0.946(82) | 0.950(28) |
| 2B | - | - | 0.984(28) | 0.886(57) | 0.836(15) |
| 3B | - | - | 0.109(36) | 0.936(69) | 0.982(40) |
| 4B | - | - | 0.927(7) | 0.993(15) | 0.992(11) |
| 5B | - | - | 0.832(15) | 0.918(62) | 0.884(32) |
| 6B | - | - | 0.972(35) | 0.992(118) | 0.989(64) |
| 7B | - | - | 0.881(21) | 0.913(61) | 0.952(37) |
| 1R | 0.993(37) | 0.667(39) | - | 0.937(109) | 0.999(5) |
| 2R | -0.933(47) | 0.814(19) | - | 0.141(92) | -0.702(16) |
| 3R | 0.867(53) | 0.688(40) | - | -0.041(95) | 0.938(8) |
| 4R | -0.988(111) | 0.681(50) | - | -0.949(243) | -0.938(106) |
| 5R | -0.955(50) | 0.939(28) | - | -0.930(179) | -0.956(76) |
| 6R | 0.750(64) | 0.861(41) | - | 0.978(184) | 0.968(77) |
| 7R | 1.000(45) | 0.934(24) | - | 0.886(151) | 0.835(59) |
